# Southern Surgical and Gynecological Association

**Published:** 1892-12

**Authors:** 


### SOUTHERN SURGICAL AND GYNECOLOGICAL ASSOCIATION.

*Fifth Annual Meeting, held in Louisville, Kentucky, November 15, 16 and 17, 1892.*

FIRST DAY—MORNING SESSION.

The Association met in the council chamber in the city hall, and was called to order at 9:30 A. M. by the President, Dr. J. McFADDEN GASTON, of Atlanta, Ga.

An address of welcome was delivered by Dr. L. S. McMurtry, of Louisville, Chairman of the Committee of Arrangements, the response to which was made by the president.

The first paper read was by Dr. Bedford Brown, of Alexandria, Va., entitled,

PERSONAL RECOLLECTIONS OF THE LATE DR. BENJ. W. DUDLEY, OF LEXINGTON, KY., AND HIS SURGICAL WORK.

The speaker paid an eloquent tribute to Dr. Dudley, and characterized him as the greatest lithotomist that this country has ever produced, and the most successful in the history of the world. The speaker's close relationship to Dr. Dudley as private pupil and assistant for two years enabled him to present a clear and faithful sketch of his character and surgical work.

EXPERIENCES IN PELVIC SURGERY.

This was the title of a paper read by Dr. A. V. L. Brokaw, of St. Louis, Mo. Of all the surgical problems difficult to solve, it may be truthfully said, that those met with in the pelvis are the most trying. The speaker knew of no surgical work which will compare with the experiencies found in the pelvis; a diversity of conditions, complications and unexpected happenings are ever presenting. In a series of many operations but few will be alike in every particular. As his experience became larger, he was free to confess his inability to correctly diagnose the character of abdominal and pelvic troubles. He had diagnosed pus tubes and found extra-uterine pregnancy; diagnosed extra-uterine pregnancy and found pus; diagnosed ovarian lesions and found the trouble located in the tubes, and *vice versa.* When well defined pelvic lesions exist, nothing short of radical measures succeed. The one condition above all others where exploratory incision should be adopted was in cases of suspected extra-uterine pregnancy. It was correct and good surgery to open the abdomen and wait for all the classical signs to appear. The symptoms of extra-uterine pregnancy were so frequently obscure and unreliable that he was firmly convinced a radical position should be taken. A case was cited in point.

DR. WILLIAM WARREN POTTER, of Buffalo, desired to indorse that portion of the paper pertaining to any early exploratory incision in cases of suspected extra-uterine pregnancy. As regards the use of the sound, he had brought an indictment against it some six or eight years ago, consequently he would not expatiate upon the subject at this time.

DR. JOSEPH TABER JOHNSON, of Washington, said that as soon as the surgeons diagnosed something in the abdominal cavity that ought not to be there, anatomically or physiologically, and was histologically wrong, it should be removed. An exploratory operation was jurisdiction in cases of suspected extra-uterine pregnancy, and the surgeon should base his further procedures upon what he finds after making the exploration.

DR. W. E. B. DAVIS, of Birmingham, Alabama, thought the pendulum relative to surgical interference had swung a little too far. He believed that a great many of the so-called "tinkerers" who succeeded in relieving their patients, did not accomplish it so much by the local treatment they used, as by having patients under their care, keeping the bowels open, giving constitutional treatment, seeing them regularly, etc. While, by so doing, they may not be cured in all cases, they were benefitted. Regarding the diagnosis, surgeons who are opening the abdomen constantly, would rarely give a positive diagnosis in the case. Dr. Davis cited the case of a woman who had an acute attack of peritonitis, and the history was the same as from pelvic abscess.

DR. BROKAW, in closing the discussion, said that in every case of suspected extra-uterine pregnancy, it was good surgery to make an exploratory incision and operate before rupture took place.

DR. CORNELIUS KOLLOCK, of Cheraw, South Carolina, read a paper on "Craniotomy Upon the Living Fœtus not Justifiable." He said this operation implied the death of the fœtus and a frightful mutilation of its body, often accompanied by serious lacerations of the vagina and adjacent tissues of the mother. Recent advances in obstetrics, gynecology and abdominal surgery contribute largely to a demonstration of the fact that a timely resort to Cæsarean section in pelvic obstruction is the great factor to success. In Germany, out of 149 cases of contracted pelvis, 109 mothers and 136 children were saved. If craniotomy had been done in those cases, 149 children would have been destroyed and probably 50 women —perhaps more, making a sacrifice of at least 199 lives. In many of these cases, exhaustion had supervened and septic influence had already been excited. This, added to a tardy disposition to union by first intention, caused by contusion of the parts involved in the uterine incision, lessened materially the woman's chances for recovery. Zweiffel was successful in twenty-nine cases out of thirty; Schauta did Cæsarean section fifteen times without a single death. Recently in eighteen operations done in Louisiana, fourteen were successful. Of eight in Ohio, six were successful. Dr. Price has done Cæsarean section a number of times successfully. Dr. Kollock is firmly convinced that eighty-five or ninety-five per cent. of the cases of obstruction of the pelvis forbidding the delivery of the fœtus in the natural way, might be saved by a timely resort to the Cæsarean section.

DR. W. D. HAGGARD, of Nashville, emphasized the position taken by Dr. Kollock. He believes that when the profession fully realizes the immense difference in the number of lives saved by Cæsarean section over craniotomy there will be no doubt as to its preference to the latter operation.

DR. HUNTER MCGUIRE, of Richmond, favored Cæsarean section. Some time ago he saw the report of a case by Dr. Thomas, of New York, where, in doing Cæsarean section, he proposed to take the uterus out of the cavity, and then open it. He thought this added very much to the danger of the operation, necessitating a larger opening, exposing the cavity of the abdomen a long time to the atmosphere, etc. He does not favor this procedure.

DR. L. S. MCMURTRY, of Louisville, said that a few years ago it would have been impossible for one to have presented the views that Dr. Kollock had without meeting with violent opposition. Cæsarean section was then regarded as an extremely heroic operation, and until recent years, the mortality therefrom was very great; but since it has been carried to the present degree of perfection by Sænger and others it has strengthened the opinions of abdominal surgeons, who now consider it preferable to craniotomy. Within the last two months symphysiotomy has been brought before the profession and practiced as an alternative in certain cases for Cæsarean section. What the future of the former operation is to be, we are not prepared to say.

DR. ARCH DIXON, of Henderson, Ky., advised Cæsarean section in a case in which he was called in consultation, but the family physician insisted upon his doing craniotomy, which was done, and while every precaution was taken with regard to rendering aseptic the field of operation, the woman developed pelvic peritonitis, and died within four days. He believed a Porro operation would have saved the life of the woman, and perhaps that of the child.

DR. W. D. HAGGARD, of Nashville, read a paper entitled :

"A Case of Extensive Hematocele Resulting from Tubal Pregnancy Rupturing into the Broad Ligament."

Although the fœtus was not found, that it was a case of tubal pregnancy with rupture into the broad ligament, is clearly established by the clinical history and post-mortem appearances summarized as follows: (1) Patient confessed having had intra-pelvic trouble previously (presumably gonorrhœa), for which she was treated locally. (2) At the time of the accident, caused by jumping from a wagon, her menses were past due. As to how long, her statements were misleading. (3) There was a fitful yet persistent flow from the uterus during her entire illness. (4) Paroxysmal, colicky pains in lower abdominal and pelvic regions of frequent occurrence. (5) Existence of a tumor above the pubes, which she probably mistook for a gravid uterus. (6) Persistent refusal to submit to a digital examination, probably fearing the detection of her pregnant state.

*Post-Mortem Appearances.*—(a) Enlarged and softened condition of the uterus with a patulous os, showing escape of a sero-sanguinal, stringy fluid. (b) Enlargement of the left tube with a well-defined cavity from which the fruit sac escaped. (c) Existence of a deciduous membrane, as revealed by the microscope. (d) Discoloration of the rectum, produced by blood dissecting around it, producing constriction and partial death.

DR. H. C. HOGAN, of Union Springs, Ala., reported a case of fibroid tumor of the uterus—pregnancy: rupture about the fourth month—operation, specimen. The woman, colored, was twenty-eight years of age, and from the symptoms and history of the case, he was satisfied there was a rupture, and the probabilities were that it was about the fourth month of gestation. He was also of the opinion that the rupture did not immediately destroy the fœtus, that it continued to grow in its abnormal position. The speaker felt sure that if he had operated on the case immediately after rupture, the patient's life would have been saved. In all cases of rupture he would advise Porro's operation to be done immediately; that in all cases, where the tumor is large or multiple, intra-mural or subperitoneal, with a sacciform dilatation of the posterior segment of the uterus, and, as above the pubic bone, or inaccessible, the same operation should be done. In all cases where the tumor is in front of the child, or blocking the passage, it should be done, provided the pregnancy has advanced to the full time, or there should be a hemorrhage. or rupture of the membranes, indicating that an abortion or miscarriage is imminent.

FIRST DAY—MORNING SESSION.

DR. GEO. A. BAXTER, of Chattanooga, Tenn., read a paper entitled "A New Operation for the Radical Cure of Inguinal Hernia." Dr. Baxter presented a radically different operation in principle from any yet given. It consists in a prolongation of the incision, after the ordinary management of the sac and after ligation, through the internal ring into a more or less extensive laparotomy as the exigencies of the case demand; lifting the neck of the sac into the abdominal opening above the ring and its fixation there by a deep suturing, cutting off the sac close above the peritoneum and its closure by buried suture, and a final closure of the abdominal opening by this and a more superficial set of sutures which pass across above the closed sac and peritoneum and underneath the deep fasciæ which are intended to approximate the homologous tissues of the abdominal wall. The ring is closed with crucial sutures dipping over cord and traversing the tissues, and the seminal canal closed with deep sutures alone.

Points of originality claimed: A line of incision suitable for any inguinal hernia, and by the fixation of the sac above the peritoneum, a deflection of all abdominal expulsive force from the ring and canal, and the thickened lining of the internal ring, and the method of closure of abdominal incision. Advantages claimed: Quick cure with avoidance of necessity of truss, deflection of expulsive force from internal opening and canal to abdominal parieties; advantage in being able to approach constriction either from without or within; avoidance of necessity for traction on sac or contents; ample room for treatment in diseased conditions of sac or contents, including gut operations if necessary.

(*To be continued.*)

